# Evaluating the Role of Autistic Traits, Social Anxiety, and Social Network Changes During Transition to First Year of University in Typically Developing Students and Students on the Autism Spectrum

**DOI:** 10.1007/s10803-020-04391-w

**Published:** 2020-02-07

**Authors:** Jiedi Lei, Mark Brosnan, Chris Ashwin, Ailsa Russell

**Affiliations:** Centre for Applied Autism Research, Department of Psychology, University of Bath, Bath, BA2 7AY UK

**Keywords:** Autism spectrum disorder, Social anxiety, Social network, Perceived social support, University, College

## Abstract

This is the first longitudinal study to quantitatively evaluate changes in social network structure (SNS) and perceived social support (PSS) amongst first-year students on the autism spectrum (n = 21) and typically developing (TD; n = 182) students transitioning to university. The relative impact of changes in SNS/PSS, students’ social anxiety and autistic traits, on first-year university transition outcomes were also examined. Both groups gained friends over time who provided better support quantity and quality during first year of university. Social anxiety showed long-term differential negative impact on students on the autism spectrum and TD students’ academic, social and personal/emotional adjustments, and institutional attachment, suggesting stakeholders should focus on delivering interventions to reduce social anxiety to improve university transition outcomes.

It has long been recognised that university transition can be a stressful time (Compas et al. 1986; Felner et al. 1983; Lambe et al. 2018; Lei et al. 2018), as students separate from established social networks at home, adjust to independent living and build new ties to integrate into the university community (Tinto 1988; Van Gennep 1960). Students who experience high levels of social anxiety (de Lijster et al. 2018), or social communication differences and a preference for sameness as exemplified by high levels of autistic traits (Jobe and Williams White 2007) may find such social network changes particularly challenging, which in turn may impact on university transition outcomes. Using a longitudinal design, the current study evaluates how changes in social network structure and perceived social support of first-year typically developing (TD) students and students on the autism spectrum^1^ might influence university transition outcomes, and to what extent these outcomes are affected by social anxiety and autistic traits.

## Social Network Changes in University Students

Social network structure (SNS) includes dimensions such as size (i.e., number of people that a person is in contact with), density (i.e., the degree of contact between network members), and composition (i.e., the relative proportion of family, friends, and other members) (Scott 2017). The functionality of social networks can be measured by perceived social support (PSS), i.e., an individual’s subjective experience of tangible (e.g., practical/informational) and less tangible (e.g., emotional/social) support provided by different network members (Cohen and Wills 1985; Roohafza et al. 2014). There has only been one previous study which simultaneously investigated changes in SNS and PSS amongst first-year TD university students finding that those who lived on campus had higher density social networks, with more friends and fewer family members compared to their peers who lived at home, and perceived friends to provide greatest support (Hays and Oxley 1986). Students who report better PSS have shown better mental and physical health (Gall et al. 2000; Tao et al. 2000). Over time, students increasingly rely on friends for informational and emotional support, and spent more leisure time with family (Friedlander et al. 2007; Hays and Oxley 1986; Swenson et al. 2008). Students who perceived greater support from professors engaged in more positive coping strategies (Tao et al. 2000), and had better mental health outcomes (Azmitia et al. 2013). The noticeable changes in PSS provided by different social network members highlights the dynamic flow of social capital within a social network over time (Azmitia et al. 2013; Friedlander et al. 2007; Gall et al. 2000; Swenson et al. 2008).

However, previous literature has some limitations. First, only one study (Hays and Oxley 1986) simultaneously measured changes in both PSS and SNS, but not beyond semester one. Second, previous measures of PSS have often asked students to report overall levels of support across general informational, emotional, and practical domains provided by the social network as a whole, rather than evaluating the unique contribution made by individual network members to the specific support domains. Therefore, it remains unclear how changes in SNS and PSS provided by different network members may contribute to university transition outcomes beyond semester one in first year.

## Autistic Traits and University Transition Outcomes

Establishing new social ties at university requires students to have sufficient social skills and confidence to approach others. However, previous studies investigating changes in SNS and PSS have not assessed relevant social factors such as social communication skills and social anxiety. Autism Spectrum Disorder (ASD) is a pervasive neurodevelopmental condition characterised by social communication difficulties and restricted and repetitive behaviours (American Psychiatric Association 2013) affecting up to 1 in 59 children (Centers for Disease Control and Prevention 2019). For many students on the autism spectrum, the inherent social communication difference not only affects their ability to establish a new functional social network at university, but also interfere with academic work such as doing group projects, and living in shared accommodation (Adreon and Durocher 2007; Gelbar et al. 2014; Lambe et al. 2018; Lei et al. 2019, 2018). Many students on the autism spectrum report high levels of anxiety (71%), loneliness (53%), and depression (47%) (Gelbar et al. 2014), as well as elevated rates of suicidal ideation and attempts (Jackson et al. 2018a) as a result of poor university adaptation.

Compared to TD students, students on the autism spectrum at university often continue to receive support from parents rather than peers (Elias and White 2018; Fleischer 2012), though to the best of our knowledge, no studies so far have directly examined the changes in SNS and PSS of students on the autism spectrum during transition to university over time. Broader autism phenotype in non-clinical populations also includes poor social communication and understanding (Austin 2005; Jobe and Williams White 2007; Sasson et al. 2013). TD students with higher levels of autistic traits (as measured by the Autism Quotient, including domains such as social skills and communication deficit, attention and switching, and lack of imagination) have reported greater loneliness, and poorer social relationship quality than their peers at university (Jobe and Williams White 2007).

## Social Anxiety and University Transition Outcomes

Another factor associated with students’ social functioning is social anxiety. Fear of negative evaluation by others, with consequent anxiety in and avoidance of social situations are key features of social anxiety disorder (Clark and Wells 1995; Rapee and Heimberg 1997). Symptoms of social anxiety affect between 19 and 23% of undergraduate TD students, (Beidel et al. 1989; Strahan and Conger 1998; Strahan 2003), and 4–29.2% of young people on the autism spectrum (Hollocks et al. 2019; Kent and Simonoff 2017). The transition to university can heighten social anxiety amongst all students, with those who do not have a clinical diagnosis for social anxiety still experiencing shyness and symptoms from time to time in various social situations at university (Purdon et al. 2001).

Prior research findings on the impact of social anxiety on students’ academic and social transition outcomes have been mixed (Arjanggi and Kusumaningsih 2016; Brook and Willoughby 2015; Strahan 2003; Zukerman et al. 2019). Some found greater social anxiety correlated with poorer academic adjustments, and suggested that highly socially anxious students may be unable to seek help for academic assignments, especially from those in a position of higher authority (e.g., teachers, tutors, or lecturers) (Arjanggi and Kusumaningsih 2016; Brook and Willoughby 2015; Zukerman et al. 2019). However, others have found that greater social anxiety did not affect academic achievement at university, and suggested students are better at coping with academic compared to social challenges (Strahan 2003).

## Current Study: Research Aims

The current study is the first to investigate how changes in SNS/PSS, autistic traits and social anxiety differentially affect first year university student transition outcomes in both typically developing and students on the autism spectrum using a longitudinal design. The study had five aims. We first evaluated changes in students’: (1) perceived distress across a range of academic, daily living, and socialization areas; (2) SNS; and (3) PSS over the first year of university. We also examined to what extent (4) changes in SNS/PSS and (5) level of social anxiety (measured over time) and autistic traits (measured at start of the academic year), influenced different first-year transition outcomes (academic, socialization, personal/emotional adjustment, and attachment to institution).

## Method

### Study Design

The current study was approved by the university’s departmental ethics committee and is in line with the Declaration of Helsinki as revised in 2000. All participants received study information and completed written informed consent online via Qualtrics prior to participating in the research study. Eligibility criteria included having attended secondary school in the UK, aged 17–19 years, and starting first-year of university in the UK for the first time. Recruitment methods included handing out flyers on university campus, posting on social media, and through presentations given in introductory lectures during the first two weeks of semester one to first-year university students.

All participants completed baseline questionnaires within the first two weeks of starting university and were re-contacted via email in December (towards the end of semester one) to complete session two, and in March (towards the end of semester two) to complete session three. All sessions were completed online via Qualtrics. At the end of each session, participants were shown an information sheet about available services both within the university, in the local area, and also national charities for mental health/autism support. For each session completed, participants were either entered into a prize draw to win a £50 gift voucher or received one course credit. Typically developing and students on the autism spectrum were recruited at the same time, and the data were analysed separately due to differences in sample sizes.

### Participants

#### Typically Developing (TD) Group

Eligibility criteria for TD students included not experiencing any current acute or chronic mental or physical health conditions or any specific learning disability at the time of study enrolment (i.e., within first two weeks of starting semester one), to ensure that the TD student group did not have any additional vulnerabilities at the start of university. A sum total of 267 TD students completed the first session, with 106 students recruited in 2017, and 259 in 2018. Overall, 182 students completed all three sessions (retention rate of 70.27%).

#### Autism Group

A total of 28 students on the autism spectrum completed the first session, with 8 students recruited in 2017, and 20 in 2018. Twenty-one students completed all three sessions (retention rate 75%). All students disclosed that they had received an autism diagnosis from a clinical professional (i.e., not self-diagnosed). Seventeen students had a clinical diagnosis of Asperger’s syndrome, 10 with ASD, and 1 with Pervasive Developmental Disorder—Not Otherwise Specified. All students were known to and have disclosed and verified their autism diagnosis by showing official diagnostic letters from clinical professionals to their university’s disability team, through which they can access various types of support on campus. Six students (21%) reported having at least one other co-occurring condition, including anxiety (n = 3), depression (n = 3), attention deficit hyperactivity disorder (n = 1), sensory processing disorder (n = 1), and dyspraxia (n = 1). Five of these six students completed all three sessions and were included in the final sample (n = 21).

### Measures

#### Autism Quotient-Short (AQ-S; Hoekstra et al. 2011)

AQ-S is a 28-item abridged version (Hoekstra et al. 2011) of the full 50-item Autism Quotient scale, a self-report measure of autistic traits. See Appendix 1 for more details. All participants completed the AQ-S at T1 to measure level of autistic traits.

#### Social Anxiety Scale: Adolescents (SAS-A; La Greca et al. 2015)

SAS-A is a 22-item self-report measure of social anxiety in adolescents (La Greca et al. 2015). Validation of the SAS-A is described by La Greca et al. (2015), and see Appendix 1 for more details. All participants completed the SAS-A at T1, T2 and T3 to monitor changes in social anxiety over time.

#### Social Network and Perceived Social Support (SNaPSS; Lei et al. 2019)

The SNaPSS is an online self-report tool to characterise perceived distress frequency across academic, daily living, and socialization areas, and SNS and PSS amongst students going to university. Details of the measure development and scoring can be found in Lei et al. (2019), and in Appendix 1. Participants completed the SNaPSS at T1, T2, and T3 to assess changes in SNS and PSS over time.

#### Student Adaptation to College Questionnaire (SACQ; Baker and Siryk 1984)

The SACQ is a 67-item self-report questionnaire evaluating students’ transition outcomes including academic, social, personal emotional adjustments, and goal commitment and institutional attachment when adapting to university life (Baker and Siryk 1984). See Appendix 1 for more details. Participants completed the SACQ at T2 and T3, to monitor changes in transition outcomes across first year of university.

### Data Analyses

All data analyses were completed using SPSS version 25 (IBM SPSS Statistics 2016), and Gephi2 (Bastian et al. 2009) to calculate SNS density and visualise social network structure. We used an alpha level of .05 and used Bonferroni corrections to adjust for multiple comparisons where appropriate. We used parametric tests for analysing data from TD students, and non-parametric test for data from students on the autism spectrum, due to the relatively smaller sample size for students on the autism spectrum (n = 21). Analyses included only students who completed the study and were completed in three steps for each study. First, we assessed changes in social anxiety over time, using either repeated measures ANOVA (TD group), or Friedman’s Test (autism group). Second, we investigated changes in perceived distress frequency, SNS and PSS over time, using either repeated measures ANOVA (TD group), or Friedman’s tests (autism group). Third, we explored how levels of autistic traits, social anxiety, as well as changes in SNS and PSS might influence different aspects of students’ transition outcomes, using either stepwise linear regressions (TD group), or Kendall’s tau-b correlations (autism group). See Appendix 2 for additional details.

## Results

### Participant Demographics

Table 1 shows participant demographic information for TD students (n = 182) and students on the autism spectrum (n = 21) who completed the study. See Appendix 3 for more details on analyses comparing demographic variables across year group and retention status. There were no differences across educational cohorts and retention status amongst TD students, or retention status amongst students on the autism spectrum across any demographic variables.

**Table 1:** Demographic information for typically developing (n = 182) and autistic (n = 21) students

	TD		ASD	
	M (SD)	Range	M (SD)	Range
Age (years)	18.27 (0.50)	17–19	18.33 (0.48)	18–19
Sex	(n)	(%)	(n)	(%)
Male	36	19.78	11	52.4
Female	146	80.22	10	47.6
A-level average score^a^	5.10 (0.56)	3–6	4.31 (1)	2.5–6
Autistic traits (AQ-S total)^b^	62.70 (9.01)	42–89	83.19 (10.32)	64–104
Social anxiety (SAS-A-total)^c^				
T1	56.08 (11.83)	24–85	71.24 (12.74)	46–89
T2	52.12 (12.88)	23–90	71.43 (12.38)	51–90
T3	51.11 (13.59)	21–90	67.24 (12.12)	46–88
Ethnicity	(n)	(%)	(n)	(%)
Caucasian	144	79.12	21	95.2
Asian	26	14.29	1	4.8
Black	3	1.65	0	0
Mixed/other	9	4.95	0	0
Degree faculty	(n)	(%)	(n)	(%)
Sciences	32	17.58	8	38.1
Technology	6	3.30	3	14.3
Engineering	8	4.40	1	4.8
Mathematics	2	1.10	1	4.8
Arts and humanities	4	2.20	1	4.8
Social sciences	130	71.43	7	33.3

*TD* typically developing, *ASD* autism spectrum disorder, *AQ-S* autism quotient-short, *SAS-A* social anxiety scale for adolescents

^a^A-level average score is measured on a scale of 6 (A*) to 1 (E)

^b^AQ-S has a recommended cut-off score of > 65

^c^SAS-A has a recommended clinical cut-off score of 50

For TD students, repeated measures ANOVA found a main effect of time for changes in social anxiety (*F*(2, 180) = 33.73, *p* < .001, η_p_^2^ = .27), with highest level of overall social anxiety symptoms reported at T1 relative to T2 (*p* < .001) and 3 (*p* < .001), and did not change between T2 and T3 (*p* = .223). For subsequent analyses, we used the mean of the total social anxiety score across T1, T2, and T3 as a control variable to reflect overall levels of social anxiety experienced by the student across the transition process, rather than taking the baseline social anxiety score by itself due to potential ceiling effects.

For students on the autism spectrum, Friedman’s test found students showed significant differences in social anxiety over time (χ^2^(2) = 8.22, *p* = .016). Post hoc analyses using Wilcoxon signed-rank test with Bonferroni to correct for multiple comparisons resulted in an adjusted alpha level of .017. Median (interquartile range) level of social anxiety across the three time points were 71 (61.5–84.5) (T1), 69 (62–82) (T2), and 65 (59–77) (T3). Social anxiety did not differ between T1 and T2 (*Z* = − .02, *p* = .985), or 3 (*Z* = -1.96, *p* = .05), though significantly decreased between T2 and T3 (*Z* = − 2.65, *p* = .008). Similar to TD students, we computed the mean level of social anxiety (T1 to T3) to be used in subsequent analyses, to avoid any potential ceiling effects at T1.

### Changes in Perceived Distress Frequency (T1 to T3)

Table 2 shows changes in perceived distress frequency for academic, daily living, and socialization areas over time for TD students and students on the autism spectrum.

**Table 2:** Changes in perceived distress frequency, social network structure, and perceived social support over time, as measured by social network and perceived social support (SNaPSS)

	TD (n = 182)						ASD (n = 21)					
	Time 1		Time 2		Time 3		Time 1		Time 2		Time 3	
	M (SD)	Range	M (SD)	Range	M (SD)	Range	M (SD)	Range	M (SD)	Range	M (SD)	Range
Perceived distress												
Academic	4.91 (3.23)	0–21	8.31 (3.94)	0–19	8.15 (4.06)	0–20	8.95 (4.81)	0–16	11.86 (4.20)	5–20	11.38 (5.58)	2–20
Daily living	5.44 (3.48)	0–18	4.88 (3.47)	0–15	4.36 (3.30)	0–16	8.52 (4.90)	1–16	8.38 (4.93)	0–17	8.14 (5.26)	0–19
Socialisation	6.44 (4.75)	0–19	4.43 (4.07)	0–18	3.82 (3.75)	0–18	9.57 (6.02)	1–20	10.53 (4.69)	3–20	9.52 (5.42)	1–20
SNS												
Size	11.98 (5.10)	0–20	10.58 (5.13)	0–20	10.33 (5.17)	0–20	8.04 (5.03)	0–20	8.19 (5.05)	1–20	7.33–5.27	0–18
Density	.36 (.19)	0–1	.31 (.16)	0–1	.30 (.16)	0–1	0.32 (0.19)	0–0.7	0.39 (0.26)	0.13–1	0.37 (0.28)	0–1
% family	37.13 (19.52)	0–100	30.66 (17.21)	0–100	30.83 (15.18)	0–75	27.21 (18.89)	0–72.73	38.58 (25.60)	0–100	27.33 (21.52)	0–90.91
% friends	60.65 (18.50)	0–100	67.54 (18.51)	0–100	67.68 (16.20)	0–100	48.53 (24.38)	0–85	54.06 (26.66)	0–100	58.64 (33.01)	0–100
% other	3.49 (7.22)	0–33.33	.70 (2.89)	0–23.53	.88 (3.76)	0–27.27	10.17 (19.60)	0–81.82	7.36 (13.97)	0–57.14	9.26–18.99	0–66.67
PSS quantity												
Academic	2.93 (2.69)	0–10.29	2.92 (1.88)	0–9	2.92 (2.06)	0–10.79	2.25 (2.62)	0–8.5	2.71 (2.25)	0–7.5	2.43 (1.91)	0–5
Daily living	4.77 (2.11)	0–10	3.76 (2.14)	0–10	3.22 (1.92)	0–10	4.26 (2.57)	0–9.9	3.78 (2.56)	0–10	3.32 (2.35)	0–8
Socialisation	4.58 (2.23)	0–10	3.32 (2.00)	0–9	2.91 (2.04)	0–8.50	3.53 (2.43)	0–8.4	3.75 (2.64)	0–10	2.83 (1.78)	0–6.33
Family	6.22 (3.31)	0–15	3.89 (2.89)	0–15	3.34 (2.74)	0–14	4.99 (4.02)	0–15	4.28 (3.8)	0–14.5	2.90 (3.02)	0–11.5
Friends	5.57 (3.28)	0–13.17	5.90 (2.99)	0–13.50	5.58 (2.89)	0–14.50	4.03 (4.17)	0–14	5.22 (3.80)	0–11	4.38 (4.06)	0–11.83
Other	0.49 (1.18)	0–6	0.21 (1.01)	0–9	0.13 (0.76)	0–8	1.02 (1.85)	0–7	0.74 (1.68)	0–6	1.30 (2.49)	0–8
PSS quality												
Academic	4.83 (3.86)	0–15	5.19 (3.09)	0–14.5	5.21 (3.13)	0–14.67	3.54 (3.64)	0–12	4.62 (3.59)	0–13	3.42 (2.77)	0–9
Daily living	7.24 (2.75)	0–15	6.77 (2.97)	0–13	6.54 (2.99)	0–13	6.42 (4.07)	0–15	5.40 (3.13)	0–10	5.16 (2.98)	0–10
Socialisation	6.84 (3.00)	0–15	5.80 (2.91)	0–13.45	5.29 (3.24)	0–12.17	4.46 (3.28)	0–10	4.45 (3.27)	0–10	4.94 (3.39)	0–14
Family	9.41 (4.10)	0–15	7.65 (4.66)	0–15	7.19 (4.79)	0–15	6.53 (4.70)	0–15	6.11 (5.00)	0–15	4.85 (4.63)	0–13.5
Friends	8.49 (4.21)	0–15	9.67 (3.79)	0–15	9.54 (3.86)	0–15	5.62 (5.06)	0–15	6.71 (4.11)	0–15	6.42 (4.98)	0–15
Other	1.01 (2.59)	0–15	.43 (1.85)	0–13	0.30 (1.43)	0–10	2.26 (3.96)	0–15	1.64 (3.91)	0–14	2.26 (4.08)	0–12

*TD* typically developing, *ASD* autism spectrum disorder, *SNS* social network structure, *PSS* perceived social support

For TD students, using repeated measures ANOVA, we found no significant main effect of time (*F*(2.56, 331.27) = 2.56, *p* = .084, η_p_^2^ = .014). A significant main effect of type (*F* (1.89, 342.47) = 64.79, *p* < .001, η_p_^2^ = .264), and a significant time by type interaction on perceived distress frequency (*F*(3.14, 568.88) = 86.88, *p* < .001, η_p_^2^ = .324) were found. TD students perceived significantly greater distress in academic areas compared to daily living (p < .001) and socialization (*p* < .001) across all three time points. In contrast for students on the autism spectrum, using Friedman’s test, there were no differences in perceived distress frequency in academic, daily living, and socialization domains over time (χ^2^(2) = 3.71, *p* = .156), nor differences in total perceived distress frequency across each time-point (χ^2^(2) = 2.33, *p* = .311).

For TD students, autistic traits did not interact with either time or type to influence any changes in perceived distress frequency. However, mean levels of social anxiety significantly interacted with type (*F*(1.89, 338.72) = 5.51, *p* = .005, *η*_p_^2^ = .03), and students with higher social anxiety perceived greater distress frequency in socialization areas compared to daily living areas (*p* = .001). In contrast for students on the autism spectrum, using Kendall’s tau-b correlation, greater mean level of social anxiety, not autism symptom severity, was associated with greater perceived distress in academic (*τ*_b_ = .32, *p* = .046), daily living (*τ*_b_ = .33, *p* = .042), and socialization (*τ*_b_ = .45, *p* = .004) areas.

### Changes in Social Network Structure (SNS) (T1 to T3)

Table 2 and Appendix 4 show changes in SNS over time for TD students and students on the autism spectrum. For TD students, repeated measures ANOVAs showed a significant main effect of time for social network size (*F*(1.72, 312.51) = 14.21, *p* < .001, η_p_^2^ = .073), density (*F*(1.89, 341.96) = 8.51, *p* < .001, η_p_^2^ = .045), and network composition of percentage of family (*F*(1.71, 309.59) = 5.25, *p* < .001, η_p_^2^ = .078), friends (*F*(1.97, 356.03) = 12.26, *p* < .001, η_p_^2^ = .096), and other network members (*F*(1.48, 267.83) = 20.71, *p* < .001, η_p_^2^ = .103). Networks had greater size and density at T1 relative to T2 (*p* < .001; *p* = .008) and T3 (*p* < .001; *p* = .001), though did not differ between T2 and T3 (*p* = .403; *p* = .878). In contrast for students on the autism spectrum, using Friedman’s test, no statistically significant differences over time were found for network size (χ^2^(2) = 0.46, *p* = .796) or density (χ^2^(2) = 0.08, *p* = .961).

For network composition, TD students reported more family and other network members at T1 relative to T2 (*p* < .001; *p* < .001) and T3 (*p* < .001; *p* = .007), though no differences between T2 and T3. TD students reported lowest percentage of friends at T1 relative to T2 (*p* < .001) and T3 (*p* < .001), though no differences between T2 and T3. Similarly, for students on the autism spectrum, the mean percentage of family, friends, and other network members across all three time-points significantly differed (χ^2^(2) = 27.71, *p* < .001). Post-hoc analyses using Wilcoxon signed-rank test with Bonferroni to correct for multiple comparisons resulted in an adjusted alpha level of 0.017. Median (interquartile range) percentages for network composition over time were 31.27% (19.92–39.39%) for family, 59.64% (31.67–69.44%) for friends, and 3.70% (0–12.68%) for other network members. Students on the autism spectrum had a significantly greater mean proportion of friends than family (*Z* = − 2.52, *p* = .012), and both a greater mean proportion of family (*Z* = − 3.56, *p* < .001) and friends (*Z* = − .384, *p* < .001) compared to other network members.

For TD students, neither autistic traits nor social anxiety interacted with time to influence changes in any SNS measure. Appendix 4a shows examples of both social pruning and network expansion observed over time in TD students.

For students on the autism spectrum, using Kendall’s tau-b correlations, neither autism symptom severity nor social anxiety were associated with mean social network size (*τ*_b_ = .06, *p* = .715; *τ*_b_ = − .08, *p* = .61, respectively), density (*τ*_b_ = − .02, *p* = .903; *τ*_b_ = .04, *p* = .785, respectively), or composition (*τ*_b_ = − .12 to − .01, *p* = .466 to .952; *τ*_b_ = − .32 to .15, *p* = .054 to .414, respectively). Appendix 4b highlights individual differences in social network structural changes over time amongst students on the autism spectrum.

### Changes in Perceived Social Support (PSS) (T1 to T3)

Table 2 shows the mean quantity and quality of PSS provided by network members and across different areas over time for TD students and students on the autism spectrum.

#### By Member Over Time

For TD students, perceived support quantity provided by network members over time showed a significant main effect of time (*F*(1.80, 324.82) = 39.50, *p* < .001, η_p_^2^ = .18), network member type (*F*(1.95, 352.22) = 433.44, *p* < .001, η_p_^2^ = .71), and time by network member interaction (*F*(3.65, 660.13) = 48.11, *p* < .001, η_p_^2^ = .21). PSS quantity was higher at T1 than T2 (*p* < .001), and T3 (*p* < .001), and at T2 than T3 (*p* = .007). Friends provided the greatest support quantity relative to family (*p* < .001), and other network members (*p* < .001), and family provided more support relative to other network members (*p* < .001). Neither autistic traits nor social anxiety interacted with time, or member status to influence changes in perceived support quantity.

For students on the autism spectrum, using Friedman’s test, we observed significant differences in students’ perceived support quantity (χ^2^(2) = 14.77, *p* = .001) provided by different network members across all three domains over time. Post-hoc analyses using Wilcoxon signed-rank test with Bonferroni to correct for multiple comparisons resulted in an adjusted alpha level of 0.017 for both support quantity. For PSS quantity over time, median (interquartile range) were 3.67 (1.89–5.44) for family members, 4.58 (2.04–6.72) for friends, and 0 (0–2.17) for other network members. Family (*Z* = − .3.53, *p* < .001) and friends (*Z* = − .3.41, *p* = .001) provided greater PSS quantity than other network members, though no differences between family and friends (*Z* = − .1.38, *p* = .167). Kendall’s tau-b correlations found no significant associations between support quantity across different network members and autism symptom severity (*τ*_b_ = 0 to 0.1, *p* = .952 to 1), or social anxiety (*τ*_b_ = − .17 to .12, *p* = .123 to .525).

For TD students, perceived support quality provided by network members over time, a significant main effect of time (*F*(1.93, 348.66) = 9.04, *p* = .002, η_p_^2^ = .03), member type (*F*(1.83, 331.45) = 570.59, *p* < .001, η_p_^2^ = .76), and time by member type interaction (*F*(3.91, 707.32) = 23.34, *p* < .001, η_p_^2^ = .11) were found. PSS quality was higher at T1 than T3 (*p* = .003), though no differences between T1 and T2, nor T2 and T3. Friends provided best quality support relative to family (*p* < .001), and other network members (*p* < .001), and family provided better quality support relative to other members (*p* < .001). Neither autistic traits nor social anxiety interacted with time or network member type to influence changes in perceived support quality.

For students on the autism spectrum, for PSS quality over time, we observed significant differences in students’ perceived quality of support (χ^2^(2) = 10.76, *p* = .005) provided by different network members across all three domains over time. Post-hoc analyses using Wilcoxon signed-rank test with Bonferroni to correct for multiple comparisons resulted in an adjusted alpha level of 0.017 for support quality. For PSS quality over time, median (interquartile range) were 5.33 (2.81–7.08) for family, 6.67 (3.83–8.40) for friends, and 0 (0–4.75) for other network members. Family (*Z* = − .3.29, *p* = .001) and friends (*Z* = − .3.10, *p* = .002) provided better quality support than other network members, though no differences between family and friends (*Z* = − .96, *p* = .339). Kendall’s tau-b correlations found no significant associations between support quantity across different network members and autism symptom severity (*τ*_b_ = − .039 to .00 *p* = .808 to 1), or social anxiety (*τ*_b_ = − .22 to .28, *p* = .19 to .83).

#### By Area Over Time

For TD students, perceived support quantity provided across different domains (academic, daily living, and socialization) over time, a significant main effect of time (*F*(1.80, 324.82) = 39.50, *p* < .001, η_p_^2^ = .18) and domain (*F*(1.88, 340.70) = 44.88, *p* < .001, η_p_^2^ = .19), and time by domain interaction were found (*F*(3.57, 645.22) = 20.46, *p* < .001, η_p_^2^ = .10). PSS quantity was greater at T1 than T2 (*p* < .001) and T3 (*p* < .001), and at T2 than T3 (*p* = .007). Participants perceived greater support quantity in daily living skills relative to academic (*p* < .001) and socialization (*p* = .003), and also greater support quantity in socialization relative to academic area (*p* < .001). Neither level of social anxiety nor autistic traits significantly interacted with time to influence changes in perceived support quantity. However, a significant interaction between level of social anxiety and support domain was found (*F*(1.88, 336.54) = 4.68, *p* = .011, η_p_^2^ = .03). TD students with greater social anxiety had greater PSS quantity in socialization than academic area (*p* = .04). No interaction between autistic traits and domains was found.

For students on the autism spectrum, using Friedman’s test, we observed significant differences in students’ perceived quantity (χ^2^(2) = 6.03, *p* = .049) of support across the three domains over time. Post-hoc analyses using Wilcoxon signed-rank test with Bonferroni to correct for multiple comparisons resulted in an adjusted alpha level of 0.017 for quantity support. For PSS quantity over time, median (interquartile range were 2.33 (0.82–4.08) for academic, 3.33 (2–5.28) for daily living, and 3.92 (1.75–4.63) for socialization areas. PSS quantity was greater in both daily living (*Z* = − .3.04, *p* = .002), and socialization (*Z* = − .2.67, *p* = .008) compared to academic studies, though no differences between daily living and socialization areas were observed (*Z* = − .946, *p* = .344). Kendall’s tau-b correlations found no significant associations between PSS quantity in any domains, with either autism symptom severity (*τ*_b_ = − .099 to .122, *p* = .36 to .54), or social anxiety (*τ*_b_ = .039 to .22, *p* = .164 to .808).

For TD students, perceived support quality provided across different domains over time, a significant main effect of time (*F*(1.93, 348.66) = 6.26, *p* = .002, η_p_^2^ = .03), domain (*F*(1.88, 339.72) = 54.79, *p* < .001, η_p_^2^ = .23), and time by domain interaction (*F*(3.68, 665.73) = 9.90, *p* < .001, η_p_^2^ = .052) were found. PSS quality was greater at T1 than T3 (*p* = .003), though no differences between T1 and T2, or T2 and T3. PSS quality was greater in daily living areas than academic (*p* < .001) or socialization (*p* < .001), and also greater in socialization relative to academic area (*p* < .001). Neither social anxiety nor autistic traits significantly interacted with time to influence changes in PSS quality. However, a significant interaction between mean level of social anxiety and domain of support was found (*F*(1.89, 337.75) = 5.15, *p* = .007, η_p_^2^ = .03). TD students with greater social anxiety perceived better socialization support than academic support (*p* = .003). No interaction between autistic traits and domains was found.

For students on the autism spectrum, using Friedman’s test, we observed significant differences in students’ perceived quality (χ^2^(2) = 9.10, *p* = .011) of support across the three domains over time. Post-hoc analyses using Wilcoxon signed-rank test with Bonferroni to correct for multiple comparisons resulted in an adjusted alpha level of 0.017 for quality of support. For PSS quality over time, median (interquartile range) were 4 (1.67–5.67) for academic, 6 (4–7.08) for daily living, and 4.18 (3.33–6.19) for socialization areas. PSS quality was better in daily living relative to academic studies (*Z* = − .2.62, *p* = .009), though no differences between socialization and academic areas (*Z* = − .1.89, *p* = .059), or daily living (*Z* = − .1.55, *p* = .121). Kendall’s tau-b correlations found no significant associations between PSS quality in any domains, with either autism symptom severity (*τ*_b_ = − .15 to .09, *p* = .063 to .348), or social anxiety (*τ*_b_ = − .15 to .07, *p* = .348 to .694, respectively).

### University Transition Outcomes

Table 3 shows transition outcomes (SACQ) at T2 and T3 for TD students and students on the autism spectrum.

**Table 3:** Students’ transition outcomes at times 2 and 3, as measured by student adaptation to college questionnaire (SACQ)

	TD (n = 182)				ASD (n = 21)			
	Time 2		Time 3		Time 2		Time 3	
	M (SD)	Range	M (SD)	Range	M (SD)	Range	M (SD)	Range
Academic	143.96 (23.12)	67–196	144.32 (31.95)	76–439	126.48 (26.87)	62–186	125.71 (29.58)	75–177
Social	128.42 (25.19)	46–178	128.01 (26.36)	39–176	97.76 (26/19)	44–132	99.14 (25.24)	55–142
Personal emotional	82.40 (18.70)	31–133	84.70 (19.46)	37–129	62.05 (17.85)	25–87	63.95 (20.24)	24–104
Attachment	108.10 (17.00)	38–135	106.87 (18.47)	39–134	87.95 (18.60)	44–117	95.29 (18.64)	51–119
Total	412.69 (59.84)	221–548	413.04 (61.83)	222–572	333.14 (72.60)	168–457	335.38 (73.95)	270–462

#### TD Students

Pearson’s correlation showed a significantly positive correlation between overall transition outcome at T2 and T3 (*r* = .78, *p* < .001), as well as academic (*r* = .50, *p* < .001), social (*r* = .79, *p* < .001), personal emotional (*r* = .70, *p* < .001), and attachment to institution (*r* = .75, *p* < .001) subscales. Paired sample t-test showed no significant differences between T2 and T3 for the total or any subscale scores when Bonferroni is used to control for multiple comparisons (*p* > .01). The average scores for SACQ total and subscales from T2 and T3 were used as dependent variables for all subsequent stepwise linear regression models, assessing how autistic traits, mean level of social anxiety, and changes in SNS and PSS might influence transition outcomes.

Both levels of social anxiety (*β* = − .51, *p* < .001) and autistic traits (*β* = − .15, *p* = .041) predicted better overall transition outcome (SACQ total), and together explained 36% of variance (*F*(2, 179) = 50.87, *p* < .001). Adding T1 SNS and PSS in step 2 did not improve overall model fit (*F*_*Change*_(4, 175) = 2.01, *p* = .095, *R*^2^ Change = .03). In step 3, adding T3 SNS and PSS significantly improve model fit (*F*_*Change*_(4, 171) = 4.13, *p* = .003, *R*^2^ Change = .05), and better overall transition outcome was associated with lower social anxiety (*β* = − .50, *p* < .001), and smaller network density at T3 (*β* = − .16, *p* = .01).

For academic, personal/emotional, and attachment to institution transition outcomes (Table 4), only lower levels of social anxiety significantly predicted better transition outcomes in each domain (*p* < .001), even when measures of SNS and PSS at T1 and T3 were added to the model in steps 2 and 3. Similarly, for socialization adjustments, both lower levels of autistic traits (*p* = .001) and social anxiety (*p* < .001) predicted better socialization at university, and no measure of SNS or PSS at T1 or T3 helped to improve model fit.

**Table 4:** Step-wise linear regressions showing how changes in social network structure and perceived social support (from time 1 to time 3), and baseline characteristics (time 1) influence transition outcomes in typically developing students (n = 182)

	Academic		Socialisation		Personal/emotional		Attachment to institution	
	B (SE)	β	B (SE)	β	B (SE)	β	B (SE)	β
Model 1								
AQ-S Tot (T1)	− .11 (.22)	− .04	− .63 (.20)	− .23*	.03 (.15)	.02	− .29 (.14)	− .16
SAS-A Tot M	− .66 (.17)	− .33*	− .87 (.15)	− .43*	− .70 (.12)	− .47*	− .56 (.11)	− .40*
* R*^2^	.12		.34		.21		.25	
* F*(*2,179*)	12.62*		45.89*		24.31*		30.42*	
Model 2								
AQ-S Tot (T1)	− .08 (.22)	− .03	− .59 (.20)	− .22*	.04 (.16)	.02	− .26 (.14)	− .14
SAS-A Tot M	− .70 (.17)	− .35*	− .85 (.15)	− .41*	− .66 (.12)	− .45*	− .55 (.11)	− .39*
Size (T1)	− .40 (.36)	− .09	.13 (.32)	.03	.20 (.25)	.06	.03 (.23)	.01
Density (T1)	− 6.38(9.25)	.05	− 16.30 (8.15)	− .13	− .81 (6.52)	− .01	− 8.94 (5.86)	− .10
PSS Qty (T1)	− .14 (.50)	− .03	− .03 (.44)	− .01	− .29 (.35)	− .10	− .30 (.32)	− .10
PSS Qlty (T1)	.56 (.37)	.18	.27 (.33)	.09	.16 (.26)	.07	.48 (.24)	.22
Δ*R*^2^	.03		.03		.01		.04	
Δ*F*(*4*, *175*)	1.37		1.79		.38		2.29	
*F*(*6*, *175*)	5.16*		16.76*		8.24*		11.96*	
Model 3								
AQ-S Tot (T1)	− .11 (.22)	− .04	− .61 (.20)	− .23*	.02 (.15)	.01	− .28 (.14)	− .15
SAS-A Tot M	− .69 (.17)	− .34*	− .84 (.15)	− .41*	− .66 (.12)	− .45*	− .54 (.11)	− .39*
Size (T1)	− .57 (.41)	− .12	.01 (.37)	.00	.05 (.29)	.02	− .16 (.26)	− .05
Density (T1)	− 2.31 (9.30)	− .02	− 13.92 (8.33)	− .11	2.04 (6.58)	.02	− 6.39 (5.87)	− .07
PSS Qty (T1)	.39 (.54)	.09	.16 (.48)	.04	.08 (.38)	.03	− .04 (.34)	− .01
PSS Qlty (T1)	.16 (.40)	.05	.08 (.36)	.03	− .06 (.28)	-.03	.23 (.25)	.13
Size (T3)	.46 (.40)	.10	.28 (.36)	.06	.39 (.28)	.11	.44 (.25)	.14
Density (T3)	− 20.91 (11.20)	− .14	− 16.27 (10.03)	− .11	− 11.50 (7.92)	-.10	− 15.26 (7.06)	− .15
PSS Qty (T3)	− .90 (.55)	− .19	− .19 (.49)	− .04	− .63 (.39)	− .18	− .28 (.35)	− .08
PSS Qlty (T3)	.62 (.38)	.19	.25 (.34)	.08	.28 (.27)	.12	.23 (.24)	.10
Δ*R*^2^	.05		.02		.04		.05	
Δ*F*(*4*, *171*)	2.77		1.25		2.35		2.29	
*F*(*10*, *171*)	4.33*		10.61*		6.04*		8.75*	

*AQ-S Tot* autism quotient-short total, *SAS-A Tot M* social anxiety scale-adolescent total mean, *PSS* perceived social support, *Qty* quantity, *Qlty* quality

*p < .01 (Bonferroni corrected for multiple comparisons)

#### Students on the Autism Spectrum

Wilcoxon signed-rank test (Bonferroni corrected for multiple comparisons, with alpha level of .0125) found no significant differences between T2 and T3 in academic (*Z* = − .1.14, *p* = .889), social (*Z* = − .47, *p* = .641), or personal emotional adjustments (*Z* = − .68, *p* = .498), or attachment to institution (*Z* = − .19, *p* = .848). We used the mean adjustment score for each transition outcome domain across T2 and T3 for subsequent analyses.

Kendall’s tau-b correlations showed autism symptom severity was not associated with academic (*τ*_b_ = − .17, *p* = .289), social (*τ*_b_ = − .07, *p* = .649), personal-emotional (*τ*_b_ = − .20, *p* = .203) adjustments, or attachment to institution (*τ*_b_ = − .11, *p* = .505). However, higher level of social anxiety was associated with poorer academic adjustment (*τ*_b_ = − .41, *p* = .009), poorer personal/emotional adjustment (*τ*_b_ = − .34, *p* = .034), and poorer attachment to institution (*τ*_b_ = − .35, *p* = .027), though not with socialization adjustment (*τ*_b_ = − .19, *p* = .226).

Next, we conducted Kendall’s tau-b correlations between measures of SNS and PSS that showed significant changes over time, and different university transition outcomes. For SNS, network composition was not significantly associated with academic (*τ*_b_ = − .06 to .17, *p* = .29 to .88), social (*τ*_b_ = − ..18 to .29, *p* = .065 to .29), personal-emotional adjustments (*τ*_b_ = -.11 to .19, *p* = .227 to .88), and attachment to institution (*τ*_b_ = − ..08 to .13, *p* = .397 to .658). For PSS, total combined support quantity and quality across different areas, network members, and over time was not associated with academic (*τ*_b_ = − ..17, *p* = .277; *τ*_b_ = − ..08, *p* = .629, respectively), social (*τ*_b_ = − ..11, *p* = .506; *τ*_b_ = − ..09, *p* = .587, respectively), personal-emotional adjustments (*τ*_b_ = − ..21, *p* = .194; *τ*_b_ = − ..03, *p* = .833, respectively), or attachment to institution (*τ*_b_ = .14, *p* = .381; *τ*_b_ = .01, *p* = .928, respectively).

## Discussion

The current study was the first to employ a longitudinal design to quantitatively evaluate changes in perceived distress frequency, SNS, and PSS amongst first-year students transitioning to university. We also assessed how these changes are associated with first-year transition outcomes and the role of autistic traits and social anxiety in both TD students and students on the autism spectrum.

### Perceived Distress Frequency

Whereas TD students perceived greatest distress in academic studies, students on the autism spectrum perceived greater distress across all areas over time. One common thread linking together the academic, daily living, and socialization areas is the necessity for maintaining and engaging in social interactions across all aspects of university life, which can be anxiety-provoking and exhausting (Anderson et al. 2017; Elias and White 2018; Jackson et al. 2018b; Van Hees et al. 2015). Similarly, TD students with higher social anxiety perceived greater distress frequency also in socialization, reflecting potentially lower social competency and greater vulnerability when coping with social changes at university (de Lijster et al. 2018).

### Changes in Social Network Structure

We found that TD students reported a reduction in their social network size and density over the first semester, and this selective social pruning is concordant with the socioemotional selectivity theory (Carstensen et al. 1999; English and Carstensen 2014), which suggests social network serves to help gather information through new network ties during times of change (e.g., transitioning to university). However, during times of stability, social network serves to maintain an individual’s social and emotional wellbeing by undergoing a selective pruning process, by only keeping network members who are considered to be close and supportive to the individual. Therefore, TD students might be selectively pruning out both old (before university) and new (since university) social network ties as they settle into university life. The decrease in social network density over time also perhaps reflect the increasing separation of a TD’s student’s peer network and family networks during university, as family members are less familiar with new social network ties that the students have met at university. Although the increasing network fragmentation might reflect increasing independence an individual has in establishing his/her new social world, it could also mean that access to social capital becomes more fragmented and specialised amongst each small cluster of network members identified in one’s social network (Scott 2017).

In contrast, students on the autism spectrum did not report any significant changes in their social network size or density over time. Social networks contained an average of around 7–8 people, and a density of 0.32–0.39. From social network literature, the average social network size lies between a tight knit support clique (5 people), and a bigger and more diverse sympathy group (12 people), both of which are considered to include mostly network members from whom the individual is likely to seek advice and support when needed (Dunbar and Spoors 1995; Hill and Dunbar 2003). Students on the autism spectrum may therefore have listed primarily people they considered closest to them (as measured by SNaPSS). From the higher education literature, both social network size and density reported is concordant with previous findings in first-year TD students who live on campus (network size 7–9 people; density 0.3–0.37) (Hays and Oxley 1986). Despite converging towards an average size and density which fall within the expected range from both social network and higher education literature, SNaPSS helped to capture and visualise the great diversity and individual differences in students’ SNS over time.

Consistent with developmental literature, both autistic and TD students reported an increase in relative percentage of peers relative to family and other network members over time, as peers provide more functional support over the course of adolescence and adulthood when young people move away from home (Lee and Goldstein 2016). The current study found students on the autism spectrum established some new relationships with same aged peers at university (Barnhill 2016; Gurbuz et al. 2019; Jackson et al. 2018a; Morrison et al. 2009). Given that prior literature found elevated levels of loneliness amongst students on the autism spectrum, it may be that they are not as satisfied with their SNS compared to TD students, and are unable to initiate social activities with peers, both of which were not directly measured and remain a future direction to be explored.

### Changes in Perceived Social Support

Consistent with prior literature, both TD students and students on the autism spectrum found friends to provide better support quantity and quality compared to other network members (Hays and Oxley 1986; Swenson et al. 2008). Over time, friends may become an increasingly important source of social support, as family members begin to provide increasingly less informational and emotional support to students (Swenson et al. 2008). Concordant with prior literature where parents reported that they continued to support students on the autism spectrum at university (Cai and Richdale 2016), students on the autism spectrum also perceived family members to provide better quality support. Despite previous literature suggesting that students on the autism spectrum receive support from institution and professionals (Gurbuz et al. 2019; Ward and Webster 2018), the current study found students on the autism spectrum did not find other network members as supportive as family and friends. It might be that students on the autism spectrum did not list as many university staff or other support members using the SNaPSS because they did not feel personally close to them or may not have kept in contact with the person overseeing their support needs at university.

Both TD students and students on the autism spectrum perceived greatest support in daily living skills (such as cooking, managing time and finances), relative to socialization and academic areas. The reduced quantity and quality of PSS in academic area is especially interesting, given that academic area was perceived to be the most distressing amongst TD students. According to the stress-buffering hypothesis, social support can only buffer stress where the type of support provided matches the source of stress itself (Cohen and Wills 1985), thus the low level of PSS in academic areas may be unable to buffer against academic distress, though direction of causation between support and distress remain to be explored in future studies.

Finally, although social anxiety did not have any impact on PSS of students on the autism spectrum, TD students who had greater social anxiety perceived more support in socialization areas relative to other areas. Given that the same group of students still perceived greatest frequency of distress in socialization areas, this suggests that PSS from network members is unable to sufficiently buffer against social distress when experienced alongside social anxiety. Students with higher social anxiety might have more negative perceptions of their own social competency regardless of the amount of external support offered (de Lijster et al. 2018), and future studies can explore the impact of negative self-perception on social competence over time.

### University Transition Outcomes

We found that changes in SNS and PSS, as well as social anxiety and autistic traits had differing impact on students’ transition outcomes. For TD students, better overall transition outcome was associated with lower social anxiety over time and having a smaller social network density by the end of semester two (Time 3), though the direction of causation is unclear. Perhaps students who are less socially anxious can selectively prune their social network to maintain only social contacts that are closest and most helpful to them. Alternatively, having a closely-knit social network can also improve flow of social capital and support, and may help to maintain a low social anxiety. Future directions can explore how students utilise their social network in relation to social anxiety through qualitative interviews, to better understand the direction of causation.

For both groups, the negative association between higher levels of social anxiety and poorer academic and personal/emotional adjustments is consistent with prior literature (Arjanggi and Kusumaningsih 2016; Brook and Willoughby 2015; Zukerman et al. 2019). Previous findings have hypothesised that greater social anxiety may restrict an individual’s access to social capital and access to information, resulting in poorer academic performance. Previous findings also found lower personal emotional adjustment is associated with greater psychological distress, poorer independence in managing one’s own emotions, and being more likely to be known to the campus psychological/counselling services (Baker and Siryk 1999). The current study found that when taking into account social anxiety, changes in SNS and PSS were not associated with either academic or personal emotional adjustments, further suggesting that some of the variance associated with poor transition outcomes in either domain explained by changes in students’ social world may be largely attributable to one’s level of social anxiety.

For social transition outcomes, we found that better social adjustment in TD students was associated with lower levels of autistic traits and social anxiety, which is concordant with prior literature that examined the broader autism phenotype in TD university students (Jobe and Williams White 2007; Trevisan and Birmingham 2016). It is interesting to note that the relationship between autistic traits and socialization adjustment held even when taking into account social anxiety symptoms and changes in SNS/PSS, suggesting that autistic traits have a negative impact on social transition outcomes beyond that of social anxiety, as well as changes in an individual’s social world. Therefore, both social anxiety and autistic traits can independently contribute towards TD students’ social vulnerability when transitioning to university. In contrast, for students on the autism spectrum, socialization adjustment was not associated with changes in SNS/PSS, autistic traits, or social anxiety. The relatively small sample of students on the autism spectrum in the current study had high levels of social anxiety as well as autistic traits, and therefore may not have provided sufficient range of scores or enough power for either factor to bear a significant association with social transition outcomes. Future studies should include a larger sample of students on the autism spectrum with high versus low levels of social anxiety, to directly compare the extent to which autistic traits and social anxiety might differentially relate to social adjustments during first year of university.

Finally, both TD students and students on the autism spectrum with greater social anxiety also experienced poorer attachment to institution, suggesting poorer commitment and/or satisfaction with their degree choice, as well as reduced satisfaction with the institution that they are attending (Baker and Siryk 1999). Previous studies suggest lower scores on attachment to institution is associated with a greater likelihood of discontinuing one’s studies at university (Baker and Siryk 1999). Therefore, stakeholders should consider targeting students’ social anxiety during transition planning for both student groups, beyond support strategies aimed at improving students’ SNS/PSS at university, to try and minimise the negative impact that social anxiety has on students’ satisfaction at university and elicit more widespread positive transition outcomes.

## Strengths, Limitations, and Future Directions

The current study has many strengths. First, we used a longitudinal design which spanned over two semesters during first year of university, thus assessing longer term transition outcomes than previous studies. Second, previous studies have often assumed a relationship between greater social anxiety and a smaller and less supportive social network without directly measuring either changes in SNS or PSS during transition to university. We used a novel online tool (SNaPSS) and found that social anxiety did not affect changes in the TD students’ SNS, though did influence PSS by increasing the amount of social support provided by network members. Therefore, a directly linear relationship between social anxiety, structural and functional aspects of social network should not be assumed.

Third, we simultaneously assessed the impact that social anxiety and autistic traits had on first-year students’ university transition (Brook and Willoughby 2015; Jobe and Williams White 2007; Strahan 2003). Whereas social anxiety is more related to fear of negative evaluation by peers and rumination of negative interpretations of social interactions (Clark and Wells 1995), poor social communication skills as manifest by high autistic traits might be associated with reduced social understanding and theory of mind ability (Baron-Cohen et al. 1985). Furthermore, autistic traits encapsulates a broader range of behaviours such as preference for routines, sensitivity to numbers and patterns, and the ability to switch flexibly between tasks (Hoekstra et al. 2011). Therefore, the current study helped to understand how social and other skills affect university transition outcomes for both TD students and students on the autism spectrum, by measuring both social anxiety and autistic traits.

The current study has a set of limitations to consider. First, we observed a very high rate of social anxiety across a majority of TD students at the start of university, before they have made adjustments to fully adapt to university life (Brook and Willoughby 2015; Purdon et al. 2001; Strahan 2003). It may be that some of the students who surpassed the clinical cut-off for social anxiety in the current may have had undiagnosed social anxiety disorder. This is especially considering that patients with social anxiety disorder often have fewer primary care visits to seek help or diagnosis due to greater social avoidance (Gross et al. 2005; Roy-Byrne and Stein 2005). Future studies can include a clinically diagnosed socially anxious group as a control group, to examine generalisability of current results in TD students to a clinical population.

Second, the current study used exclusively subjective self-report measures to gain insight into first-hand experiences of life at university. However, the study lacks an objective measure of transition outcome (e.g., academic records, participation in societies/clubs/other campus events, retention rate etc.). Objective outcome measures can help assess whether perceived distress at university is due to objectively poor performance or related to trait anxiety that may have caused the participant to perceive the transition experience in a more negative manner. Future studies assessing predictors of university transition outcomes can use both subjective and objective measures.

Third, the sample of students on the autism spectrum was relatively small (n = 21). Given the longitudinal nature of the study and the need for students to complete baseline measures within first two weeks of starting university, recruitment was particularly challenging in finding first-year students on the autism spectrum who were willing to take part in research during a particularly stressful time. Recruitment challenge further highlights the need for collaboration between institutions and researchers, to ensure incoming students on the autism spectrum who are willing to take part in research have the necessary information to help them contribute.

Fourth, the current study only included a TD student group who had no current mental, physical health, or specific learning disabilities to represent students who did not experience additional vulnerabilities at the start of university. Although we still observed elevated levels of social anxiety amongst the TD students in the current study who did not have a clinical diagnosis, such selection to exclude students not on the autism spectrum who had concurrent clinical diagnoses may not be truly representative of the population of students not on the autism spectrum at university. Future studies can benefit from replicating the current study with the addition of a student group who face non-autism related vulnerabilities (such as those who have a clinical diagnosis for mental or chronic physical health condition, and/or specific learning disabilities) as an additional comparison group, to examine both the reproducibility of current results, but also highlight whether results noted in the current study are unique to students on the autism spectrum, or shared amongst more vulnerable students transitioning to university in general.

Finally, given that there is a large diversity in students’ SNS over time, it will be helpful to assess to what extent the visual presentation of social networks based on their reports are in line with students’ more abstract considerations of what their social world is like, and how satisfied they are with their social network. The relationship between SNS, PSS, and mental health may not be linear. Understanding students’ perceptions of their SNS can help stakeholders better interpret what resilience and vulnerabilities in social networks might look like for TD and students on the autism spectrum, and to help plan more tailored support to address those needs during transition to university.

## Conclusions and Practical Implications

In conclusion, our study showed the SNaPSS helped to successfully capture individual differences in SNS and PSS over time. Collecting students’ social network maps upon entering university can also help stakeholders easily visualise the current support structure that the student perceives to be most important to them, and identify which social capital resources might no longer be available to students when transitioning to university, to better focus on meeting students’ support needs in those areas. Stakeholders can also provide better training and communication between family members, peers, and university staff to further triangulate support for university students. The current study also found both autistic traits and social anxiety can impact transition outcomes for students on the autism spectrum and TD students during first year of university. Therefore, stakeholders may consider delivering workshops to help students mitigate social distress and introduce more positive coping mechanisms in managing social anxiety in the first semester of university, which might have more widespread long-term benefits in improving students’ transition outcomes across academic, social, personal-emotional domains, and increase students’ satisfaction with their degree and institution.

## Figures and Tables

**Fig. 1:**
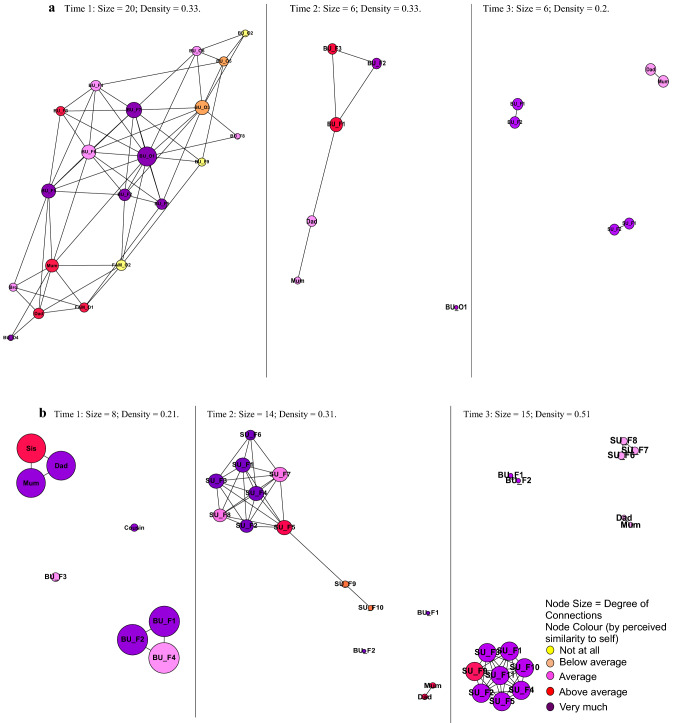
Contrasting examples of typically developing students’ social network structural changes over time, highlighting individual differences. *Bro* brother, *Sis* sister, *BU* before university, *SU* Since University, *F* friend, *O* other. **a** Participant showed decreases in both network size and density over time. **b** Participant showed increases in both network size and density over time

**Fig. 2:**
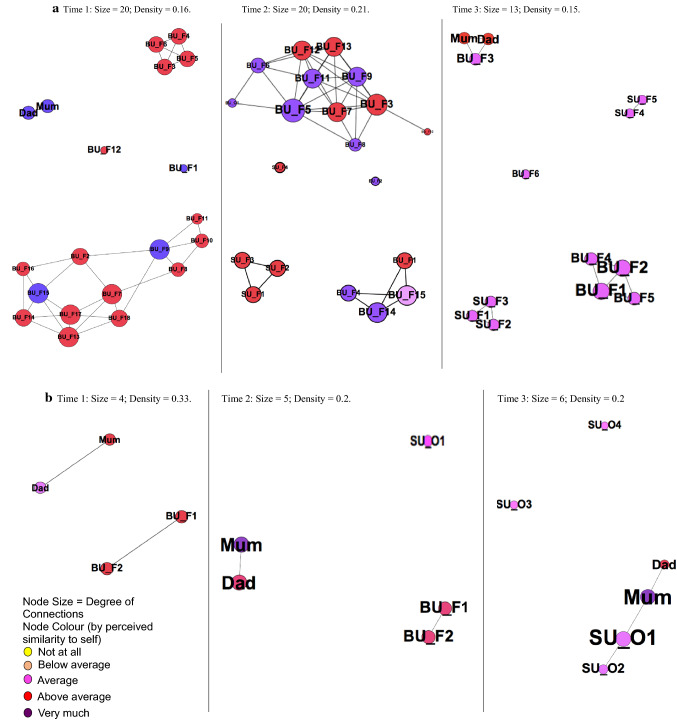
Contrasting examples of autistic students’ social network structural changes over time, highlighting individual differences. *Bro* brother, *Sis* sister, *BU* before university, *SU* since university, *F* friend, *O* other. **a** Participant showed decreases in both network size and density over tim**e. b** Participant showed increases in both network size and density over time

## Appendix 1

### Measures

#### Autism Quotient-Short (AQ-S; Hoekstra et al. 2011)

AQ-S is a 28-item abridged version (Hoekstra et al. 2011) of the full 50-item Autism Quotient scale, and has been validated in 3 independent samples across the Netherlands and UK. The abridged scale includes items that assess a range of social behaviours that are related to autistic traits, such as “I prefer to do things the same way over and over again”; “I frequently get strongly absorbed in one thing”. The abridged scale has good internal consistency (Cronbach’s alpha is between .77 and .86). The abridged AQ-S also had high predictive validity, where scores > 65 had a sensitivity of .97 and specificity of .82. Each item is rated on a four-point Likert scale, ranging from Definitely Agree (1) to Definitely Disagree (4). All participants completed the AQ-S at T1, as part of participant characterisation on level of autistic traits.

#### Social Anxiety Scale-Adolescents (SAS-A; La Greca et al. 2015)

SAS-A is a 22-item self-report measure of social anxiety in adolescents (La Greca et al. 2015), where each item is rated on a five-point Likert scale, ranging from 1 (Not at all) to 5 (All the time). Of the 22 items, three subscales are derived from 18 items, with the remaining 4 items being filler items. The three subscales consist of: (1) fear of negative evaluation (FNE; 8 items); (2) social avoidance and distress in new situations (SAD-NEW; 6 items); (3) generalised social avoidance and distress (SAD-G; 4 items). Validation of the SAS-A is described by La Greca et al. (2015). All participants completed the SAS-A at T1, as part of participant characterisation, as well as at T2 and T3 to monitor changes over time.

#### Social Network and Perceived Social Support (SNaPSS; Lei et al. 2019)

The SNaPSS is in three sections. Part one measures participants’ perceived distress frequency across 15 academic, daily living, and socialization areas on a 5-point scale ranging from 0 (never) to 4 (6 or more times a week). Part two measures SNS, and participants are asked to name up to 20 individuals (network size) with whom they have been in contact with over the past three months, and whose relationships were considered to be particularly important and worthwhile to the participant. Participants report the type of relationship (e.g., family, friends, other individuals such as teacher/lecturer, support/social worker etc.; % network composition), degree of similarity, the frequency, and modes of contact between self and each network member named. Network density is approximated by asking individuals to state whether to the best of their knowledge, each network member named know and are in contact with other network members named. Density is scored between 0 (low) to 1 (high), with high density reflecting that all network members named know and are in contact with each other. Part three measures PSS, where participants rate whether each network member named has provided them with support across any of the 15 academic, daily living, and socialization areas, and the perceived quantity and quality of support provided. Total perceived frequency and quality of support are scored between 0 and 15, with 0–5 within each of academic, daily living, and socialization domain.

#### Student Adaptation to College Questionnaire (SACQ; Baker and Siryk 1984)

The SACQ is a 67-item self-report questionnaire evaluating students’ transition outcome when adapting to university life (Baker and Siryk 1984). There are four subscales: academic adjustment (24 items), social adjustment (20 items), personal emotional adjustment (15 items), goal commitment and institutional attachment (15 items). Each item is rated on a nine-point scale ranging from “applies very closely to me” to “doesn’t apply to me at all”. The SACQ has been shown to have high internal consistency (Cronbach’s alpha ranges from .77 to .95), and has been shown to be negatively associated with measures of loneliness and anxiety, as well as positively associated with self-appraisal and positive attitudes towards family and the institution (Baker and Siryk 1999). Participants completed the SACQ at T2 and T3, to monitor changes in perceived adaptation to university life during the first year of university.

## Appendix 2

### Data Analyses

#### Typically Developing (TD) Students

First, we conducted independent sample t-tests to examine whether there are any cohort effects when comparing TD students who enrolled in 2017 versus 2018 across any baseline demographic factors (age, A-level average score, level of autistic traits and social anxiety, perceived distress frequency), as well as baseline social network structure (size, density, network composition), and overall quantity and quality of perceived social support. Next, we used chi-squared test to assess whether there are any associations between year of participation and retention status. Finally, we assessed whether across the entire sample, if there are any significant differences across the same variables as mentioned above when comparing students who dropped out of the study versus those who remained in the study.

All remaining analyses are completed using the final sample of TD students (n = 188) who completed all three questionnaire sessions online. We conducted repeated measures ANOVA to investigate changes in perceived distress frequency over time, as well as changes in SNS and PSS over time. We then conducted repeated measures ANCOVA with both social anxiety and autistic traits as covariates, to examine any interaction effects between the covariates and effect of time, type of support, or membership status. Where sphericity is violated (*p* < .05), we used Greenhouse–Geisser estimates in our reported results. We also used Bonferroni to control for multiple comparisons.

To assess whether baseline levels of autistic traits or mean level of social anxiety had any significant effect on transition outcomes (SACQ), we conducted the following analyses. First, we assessed whether there are significant differences in transition outcomes by assessing the total and subscale scores of the SACQ between times 2 and 3 by using paired sample t-test (using Bonferroni to control for multiple comparison), and also conducted Pearson’s correlation to assess the degree of similarity in ratings across the two timepoints. Next, we took an average score of the SACQ total and subscale scores measured at times 2 and 3 as the final transition outcome score. We computed step-wise linear regressions to assess how baseline levels of anxiety and autistic traits, as well as changes in social network structure and perceived social support may have had a significant impact on final transition outcomes. We entered the SACQ total and subscale scores as dependent variables in separate regression models. In step 1, we entered both the mean total social anxiety across T1 to T3 (SAS-A) and autistic trait (AQ-S) scores measured at T1 as predictors. In step 2, we entered time 1 social network size, density, and total perceived quantity and quality of social support as control variables. In step 3, we entered the same measures as step 2 that were reported at time 3 as predictors, to assess whether changes in social network structure and perceived social support over the course of transitioning to university had any impact on transition outcomes.

#### Students on the Autism Spectrum

Unlike the TD analyses, given the small sample size of students on the autism spectrum, we conducted mostly exploratory analyse using non-parametric tests. First, we conducted Mann Whitney’s U test to compare students who dropped out (n = 7) and students who completed (n = 21) the study across demographic variables, such as autism symptom severity and levels of social anxiety at baseline, as well as age, pre-university entry academic performance (average A-Level score). For students who remained in the study, we also conducted a Friedman’s test to assess changes in their level of social anxiety over time, and used Wilcoxon signed-rank test as a post hoc analysis.

All remaining analyses were conducted using the final sample (n = 21) who completed all three questionnaire sessions online and was conducted in four steps. In step one, we conducted Friedman’s test to assess whether there were any significant differences in the mean level of perceived distress frequency across time between academic, daily living, and socialization areas. We then conducted Friedman’s test to assess whether there were any differences in the total level of perceived distress frequency (the sum total of academic, daily living, and socialization perceived distress frequency at each time point) across time. We conducted Wilcoxon signed-rank test as a post hoc analysis to follow up any significant differences identified from Friedman’s tests. To examine whether autism symptom severity and level of social anxiety were associated with the mean level of perceived distress frequency for academic, daily living, and socialization areas over time, we conducted Kendall’s tau-b correlations.

In step two, for SNS, we used Friedman’s tests to assess changes in social network size and density over time. For network composition, we used Friedman’s test to examine differences in the mean percentage composition of family, friends, and other network members across all three timepoints. In step three, for PSS, we used Friedman’s tests to first examine whether there were any significant differences between the mean quantity and quality of support for academic, daily living, and socialization support provided by all network members across time. Next, we used Friedman’s tests to examine whether there were any significant differences between the mean quantity and quality of support across all three domains (academic, daily living, and socialization) across different types of network members (family, friends, and other). For both changes in SNS and PSS, we conducted Wilcoxon signed-rank test as a post hoc analysis to follow up any significant differences identified from Friedman’s tests. We also conducted Kendall’s tau-b correlations to examine whether autism symptom severity and level of social anxiety were associated with changes in SNS and PSS.

Finally, in step four, we explored the relationship between any changes identified in steps one to three across perceived distress frequency, changes in SNS and PSS, and different university transition outcomes measured at timepoints two and three. We first conducted a Wilcoxon signed-rank test to examine whether there were any significant changes in any transition outcomes between timepoints two and three, and a mean transition outcome score was computed for any domains which did not show any significant changes over time. Next, we conducted Kendall’s tau-b correlations to assess whether autism symptom severity and level of social anxiety had any significant associations with the mean score for transition outcomes across academic, socialization, personal-emotional, or attachment to institution domains. Next, we conducted separate Kendall’s tau-b correlations to examine whether any significant changes identified in steps one to three across perceived distress frequency, changes in SNS and PSS had significant associations with any of the transition outcome domains.

## Appendix 3

### Results: Participant Demographics

The current study had differences in sex ratio between TD students (19.8% male) and students on the autism spectrum (52.4% male). For the TD group, the strong bias towards females was due to the majority of students (130 out of 182) studying psychology degree (a predominantly female heavy subject). For the ASD group, the 1:1 male to female ratio observed in the current study is not dissimilar to other research projects completed in adults and young people on the autism spectrum (e.g., Jackson et al. 2018a: N = 56, 46.4% male; Gurbuz et al. 2019: ASD n = 26, 53.8% male; Anderson et al. 2018: N = 48, 50% male), suggesting that adult females on the autism spectrum may be more likely and willing to take part in research than male counterparts.

#### Typically Developing (TD) Students

To assess cohort effects by comparing TD students who enrolled in 2017 (n = 106) and 2018 (n = 153), independent sample t-tests showed that no significant differences were observed for any student demographic variables including age (*t*(257) = − 1.29, *p* = .257), pre-university entry level (A-Level average score) (*t*(256) = − .81, *p* = .418), level of autistic traits (*t*(257) = -1.41, *p* = .159), level of social anxiety (*t*(257) = .32, *p* = .747), or perceived distress frequency across academic (*t*(257) = − .03, *p* = .974), daily living (*t*(257) = .15, *p* = .885), nor socialization (*t*(257) = − .38, *p* = .708) areas. No differences in social network structure were observed for network size (*t*(257) = -1.69, *p* = .092), density (*t*(257) = − 58, *p* = .564), percentage of family (*t*(257) = .30, *p* = .767), friends (*t*(257) = -.61, *p* = .543), or other members (*t*(257) = − 1.15, *p* = .252). No differences were observed in baseline perceived overall quantity of support (*t*(257) = .17, *p* = .868), or quality of support (*t*(257) = − .11, *p* = .915).

Comparing study retention rates across 2017 (69.8%) and 2018 (70.6%), chi-squared showed that there were no associations between year of participation and study retention (χ^2^(1) = .018, *p* = .893). Overall, comparing students who completed the research study (n = 182) versus those who dropped out of the study (n = 77), independent sample t-tests showed that no significant differences were observed for any student demographic variables including age (*t*(257) =  .24, *p* = .808), pre-university entry level (A-Level average score) (*t*(256) = − .65, *p* = .519), level of autistic traits (*t*(257) = − 1.29, *p* = .199), level of social anxiety (*t*(257) = − .26, *p* = .793), or perceived distress frequency across academic (*t*(257) = 1.42, *p* = .158), daily living (*t*(257) = .03, *p* = .974), nor socialization (*t*(257) = .85, *p* = .394) areas. No differences in social network structure were observed for network size (*t*(257) =  .85, *p* = .396), density (*t*(257) = − .10, *p* = .917), percentage of family (*t*(257) =  − 1.71, *p* = .089), friends (*t*(257) = 1.56, *p* = .12), or other members (*t*(257) =  .47, *p* = .637). No differences were observed in baseline perceived overall quantity of support (*t*(257) =  .93, *p* = .078), or quality of support (*t*(257) =  − 1.30, *p* = .195).

It should be noted that the mean total score for social anxiety (SAS-A total) showed overall elevated levels of social anxiety across the sample (56.08), with a total of 115 out of 182 students scoring above the recommended clinical cut-off score of 50 at baseline for social anxiety. Given that the baseline measure was taken within the first two to three weeks of starting semester one at university amongst first year students, there may be ceiling effects as the first few weeks of university transition might be a particularly stressful and anxiety provoking time for students, relative to the rest of the academic year. We chose to conduct all subsequent analyses across the whole sample (n = 182), rather than splitting our sample into those with high versus low social anxiety at baseline. This is because of two main reasons. First, given that none of the participants had a current diagnosis of anxiety disorders at the point of enrolment, the recommended cut-off score of 50 cannot be solely taken as a clinical cut-off score, but rather to show that the elevated levels of social anxiety observed at baseline may reflect state anxiety, highlighting that experiences of elevated levels of social anxiety during the first three weeks of starting university can be pervasive across students. Second, using the whole sample enabled us to examine how baseline individual differences across a wide range of autistic traits and social anxiety might influence changes in SNS/PSS and transition outcomes, which is in line with our main research interest defined pre-hoc, rather than focusing on directly comparing those with high versus low levels of social anxiety at baseline (post-hoc).

#### Students on the Autism Spectrum

Using Mann–Whitney’s U test, we did not observe any differences between students on the autism spectrum who completed the study (n = 21), and students who dropped out (n = 7) in age (*U* = 70, *p* = .819), pre-university academic performance (average A-Level score; *U* = 64, *p* = .64), autism symptom severity (*U* = 67, *p* = .756), and social anxiety (*U* = 60, *p* = .499).

## Appendix 4a

See Fig. 1.

## Appendix 4b

See Fig. 2.

## Footnotes

1 This article chooses to use identity first language by referring to individuals as being on the autism spectrum, in line with recent research by Kenny et al. (2016) which found that identity first language is preferred by members of the autism community.
